# Physiological and transcriptomic analyses reveal the regulatory mechanisms of *Anoectochilus roxburghii* in response to high-temperature stress

**DOI:** 10.1186/s12870-024-05088-3

**Published:** 2024-06-20

**Authors:** Linghui Zhang, Heyue Yang, Mengxia Zheng, Guo Zhou, Yuesheng Yang, Siwen Liu

**Affiliations:** 1 State Key Laboratory for Conservation and Utilization of Subtropical Agro-bioresources, South China Agricultural University, Guangzhou, 510642 China; 2 Guangdong Key Laboratory for Innovative Development and Utilization of Forest Plant Germplasm, Guangzhou, 510642 China; 3 Guangdong Province Research Center of Woody Forage Engineering Technology, Guangzhou, 510642 China; 4 College of Forestry and Landscape Architecture, South China Agricultural University, Guangzhou, 510642 China; 5 Southern Medicine Research Institute of Yunfu, Yunfu, China; 6 Heny Fok School of Biology and Agriculture, ShaoGuan University, Shaoguan, 512005 China

**Keywords:** *Anoectochilus Roxburghii*, Differentially expressed genes (DEGs), High temperatures, Physiological characteristic, RNA-seq

## Abstract

**Background:**

High temperatures significantly affect the growth, development, and yield of plants. *Anoectochilus roxburghii* prefers a cool and humid environment, intolerant of high temperatures. It is necessary to enhance the heat tolerance of *A. roxburghii* and breed heat-tolerant varieties. Therefore, we studied the physiological indexes and transcriptome of *A. roxburghii* under different times of high-temperature stress treatments.

**Results:**

Under high-temperature stress, proline (Pro), H_2_O_2_ content increased, then decreased, then increased again, catalase (CAT) activity increased continuously, peroxidase (POD) activity decreased rapidly, then increased, then decreased again, superoxide dismutase (SOD) activity, malondialdehyde (MDA), and soluble sugars (SS) content all decreased, then increased, and chlorophyll and soluble proteins (SP) content increased, then decreased. Transcriptomic investigation indicated that a total of 2740 DEGs were identified and numerous DEGs were notably enriched for “Plant-pathogen interaction” and “Plant hormone signal transduction”. We identified a total of 32 genes in these two pathways that may be the key genes for resistance to high-temperature stress in *A. roxburghii*.

**Conclusions:**

To sum up, the results of this study provide a reference for the molecular regulation of *A. roxburghii*’s tolerance to high temperatures, which is useful for further cultivation of high-temperature-tolerant *A. roxburghii* varieties.

## Introduction

*Anoectochilus roxburghii* (Wall.) Lindl. (*A. roxburghii*) is an Orchidaceae family perennial plant [1]. It is a classical traditional Chinese medicine (TCM) that has been used successfully in China to treat conditions including diabetes, liver disease, and other illnesses [2]. *A. roxburghii* is primarily found in tropical climates, ranging from southeast Asia to Hawaii and extending from India over the Himalayas [3]. As one of the most valuable medicinal plants, *A. roxburghii* has seen extensive usage in the fields of medicine, functional food, cosmetics, and other areas in recent years [4]. *A. roxburghii* likes cool and moist environments, suitable for growing temperature degrees of 18–25 ℃ [5]. However, in today’s world of frequent climate extremes and increasing global warming, *A. roxburghii* is difficult to pass the summer due to high temperatures. And high temperatures severely limit the development of the *A. roxburghii* industry. Enhancing the ability of *A. roxburghii* to withstand heat and cultivating heat-resistant varieties of the organism is crucial.

High-temperature is one of the primary abiotic stresses that restrict plant growth and productivity [6]. In the past few decades, the rise in air temperature has been increased by global warming and plant growth, development, and yield have been significantly affected [7, 8]. Being sessile creatures, plants are unable to tolerate extremely high temperatures, so they have developed a sophisticated system of physiological and molecular regulatory systems to withstand heat [9, 10]. Plant heat tolerance is primarily mediated by a signal cascade, a transcription factor regulatory network, and the production of heat shock proteins [11]. Heat stress can be effectively decreased by these pathway responses, which include a rise in proline (Pro), soluble sugars (SS), free amino acids, malondialdehyde (MDA), and catalase (CAT), as well as an increase in the activity of enzymes that scavenge free radicals, such as catalase (CAT), peroxidase (POD), and superoxide dismutase (SOD) [12, 13]. Plants activate a large number of signal transduction pathways at high temperatures. According to reports, a key component of plant stimulation responses is the mitogen-activated protein kinase (MAPK) signal transduction cascade [14]. Plants with overexpression of ZmMAPK1 showed enhanced tolerance to heat stress by having more proline and lower malondialdehyde concentrations [15]. In addition, Heat shock transcription factors are critical in strengthening plant stress resistance, particularly heat resistance [16–18]. They stimulate the heat stress response by initiating the production of heat shock proteins (HSPs) [19]. HSPs can act as a molecular chaperone, inhibiting protein folding and degeneration while also increasing stress tolerance [20].

Heat stress response mechanisms in plants have attracted a lot of interest in the past several years [21]. However, most research on *A. roxburghii* has been focused on cultivation and seedling breeding, with little attention paid to the mechanism of heat tolerance [5]. In this research, we examined the physiological characteristics of *A. roxburghii* at 40 °C and analyzed its transcriptomics in response to high temperatures. These findings contribute to a deeper understanding of the physiological strategies and molecular mechanisms of *A. roxburghii* in response to high-temperature stress. And the excavation of key genes for heat tolerance is favorable to breeding high-quality *A. roxburghii* varieties with higher tolerance to high temperatures.

## Materials and methods

### Plant material and treatment

The *A. roxburghii* used in this experiment were asexual plants obtained by tissue culture and grown in the herbal garden on the campus of South China Agricultural University in Guangzhou, China.

Before the experiment began, plants were placed in a 3:1 mixture of peat soil and vermiculite in a substrate soil and incubated for 1 week in an incubator with 16 h light/8 h dark, at 25 °C.

The plants were treated with high-temperature (40 °C) of 0 (CK), 1, 2, and 4 h, and the third and fourth leaves were clipped from top to bottom. 12 plants were treated with three biological replicates of each treatment. In preparation for later research, samples were instantly frozen in liquid nitrogen and kept in an ultra-temperature refrigerator at -80 °C.

### Physiological measurements

Measurements were made during the 0 (CK), 1, 2, and 4 h heat treatment periods to compare the physiological responses of *A. roxburghii* under control and heat stress treatments. This experiment was carried out following the instructions provided by the reagent kit supplied by Suzhou Comin Biotechnology Co., Ltd. The Catalase (CAT) Activity Assay Kit (CAT-1-W; Comin, Suzhou, China), Peroxidase (POD) Activity Assay Kit (POD-1-Y; Comin, Suzhou, China), Superoxide Dismutase (SOD) Activity Assay Kit (SOD-1-W; Comin, Suzhou, China), Proline (Pro) Content Assay Kit (PRO-1-Y; Comin, Suzhou, China), BCA Protein Assay Kit (BCAP-1-W; Comin, Suzhou, China), Malondialdehyde (MDA) Content Assay Kit (MDA-1-Y; Comin, Suzhou, China), Chlorophyll Assay Kit (CPL-1-G; Comin, Suzhou, China), Hydrogen Peroxide (H_2_O_2_) Content Assay Kit (H_2_O_2_-1-Y; Comin, Suzhou, China) and Plant Soluble Sugar (SS) Content Assay Kit (KT-1-Y; Comin, Suzhou, China) were selected and used for subsequent Enzyme-linked immunoassay (ELISA) measurements. For every sample, three biological replicates were evaluated.

### Total RNA extraction, library preparation, transcriptome sequencing, and assembly

The RNAprep Pure Polysaccharide Polyphenol Plant Total RNA Extraction Kit (DP441, TIANGEN, Beijing, China) was utilized to extract total RNA. The purity, concentration, and integrity of the RNA samples were tested using NanoDrop 2000 Spectrophotometer (Thermo Scientific), and Agilent 2100 Bioanalyzer (Agilent Technologies, Palo Alto, CA, USA). After enriching Poly (A) mRNA with Oligo (dT) magnetic beads, the mRNA was randomly disrupted by the addition of Fragmentation Buffer. Following that, the first and second strands of cDNA were generated utilizing mRNA as a template, and cDNA purification was carried out. After end-repair, A-tail, and sequencing junction attachment, AMPure XP beads are used to select fragment sizes for the purified double-stranded cDNA. Lastly, PCR was used to enrich the cDNA library. Sequencing of cDNA libraries using Illumina high-throughput sequencing platform. Following the collection of high-quality sequencing data, sequence assembly was carried out. Using Trinity (version 2.14.0) software (https://trinityrnaseq.sourceforge.net), reads were first broken down into shorter fragment K-mers, and then these small fragments were extended to assemble fragments without N (Contig), and the fragment component was obtained by using the overlap between these fragments. By employing the De Bruijn plot technique and sequencing, the transcript sequences were ultimately discovered individually in each fragment component.

### Identification of DEGs and unigene functional annotation

In this study, using the DESeq2 (version 1.30.1) program, differentially expressed genes (DEGs) were screened according to the Count value of genes in each sample. |log_2_Fold Change| ≥ 2 and FDR < 0.01 were used as screening criteria to determine the significance of gene expression differences by referring to the false discovery rate (FDR). Using DIAMOND (v2.0.4) software, the unigene sequence was compared with the following databases: Non-Redundant Protein Sequence Database (NR), Swiss-Prot, KEGG, COG, EuKaryotic Orthologous Groups (KOG), Evolutionary Genealogy of Genes v4.5 (eggNOG v4.5), and Kyoto Encyclopedia of Genes and Genomes (KEGG), to obtain the protein that has the highest sequence similarity with unigene. KOBAS was used to obtain the KEGG Orthology results of the unigene in KEGG. The GO Orthology results of the new genes were analyzed using the InterPro integrated database, and after estimating the unigene’s amino acid sequence, the HMMER (v3.1b2, e-value < 1e-10) software was used to compare the unigene’s annotation information with the Pfam database.

### qRT‑PCR analysis of DEGs

To confirm the RNA-seq results further, we chose several DEGs for qRT-PCR validation. The NCBI online primer-blast was used to create specific primers for the CDS sequences of 12 DEGs. Reverse transcription of RNA samples into cDNA for qPCR using HiScript III RT SuperMix for qPCR (+ gDNA wiper) (Vazyme, Nanjing, China). Roche LightCycler® 480 Software (Roche, Basel, Switzerland) and ChamQ Universal SYBR qPCR Master Mix (Vazyme, Nanjing, China) were used for real-time quantitative PCR. *ACT2* was used as an internal reference and the 2^−ΔΔCT^ method was applied to determine the relative expression of genes.

### Statistical analyses

Analysis of data collected from physiological experiments and qRT-PCR using GraphPad Prism 9 (San Diego, CA, USA) and Microsoft Office Excel (Microsoft Corp., Redmond, WA, USA). IBM SPSS 25.0 (Armonk, NY, USA) test was used to assess the significance of differences. For multiple comparisons, use the least significant difference (LSD) approach. *P* < 0.05 was considered statistically significant.

## Results

### Physiological responses change by heat stress

**Fig. 1:**
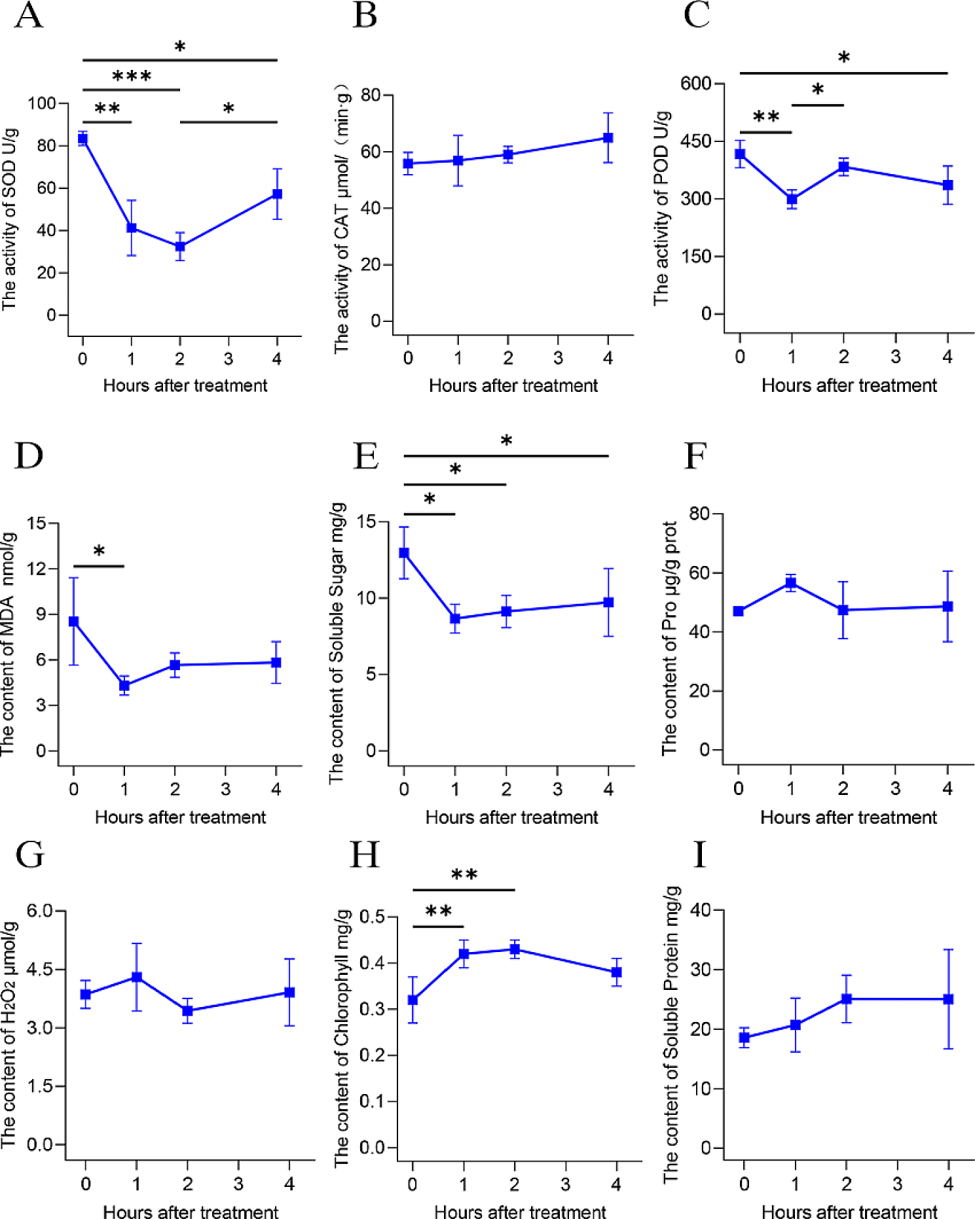
Physiological index measurement (A-I). (**A**) Changes in SOD activity, (**B**) CAT activity, (**C**) POD activity, (**D**) MDA content, (**E**) soluble sugar content, (**F**) Pro content, (**G**) H_2_O_2_ content, (**H**) chlorophyll content, and (**I**) soluble proteins content of the *A. roxburghii* under different periods of high-temperature treatment. Each data point represents the mean (± SD) of three separate experiments. The least significant difference (LSD) was used for mean comparisons, and asterisk means significantly different at *P* < 0.05 (*), *P* < 0.01 (**) and *P* < 0.001 (***). Error bars represent the SD

We analyzed the changes in the physiological responses of *A. roxburghii* under high-temperature stress. In the treatment at high temperatures, the trends of Pro and H_2_O_2_ contents in *A. roxburghii* were very complex compared with the control high-temperature stress at 0 h. Both displayed a pattern of gradual increase followed by a decline (Fig. 1F and G). Under high-temperature stress, CAT activity in *A. roxburghii* continued to grow, SOD activity showed a trend of rapid decrease and then increase, while the trend of POD activity also showed a more complex change, it decreased quickly and then increased and then decreased (Fig. 1A and C). During high-temperature stress, the MDA content of *A. roxburghii* showed a trend of rapid decline and then rise, although it slowed down after 2 h of high-temperature stress, which may be a result of the continuous accumulation of intracellular anti-reactive oxygenase during this period (Fig. 1D). In addition, SS content decreased and then slowly increased during high-temperature stress (Fig. 1E). Both chlorophyll and SP content grew and then reduced, but the decrease in chlorophyll content was more dramatic (Fig. 1H and I).

### Transcriptome quality control and DEG analysis

To determine what molecular mechanisms behind *A. roxburghii*’s response to heat stress, we compared the transcriptome profiles of *A. roxburghii* at different periods of high-temperature stress 0, 1, 2, and 4 h (H1, H2, H3, and H4 correspond to different periods). Using the Illumina high-throughput sequencing platform, the cDNA libraries of the samples were sequenced, producing a significant amount of raw data. After quality control and filtering, a total of 85.39 Gb of Clean Data was obtained, with 6.31 Gb of Clean Data per sample, a total of 285,299,193 clean paired-end reads, the percentage of Q30 bases of each sample was no less than 93.46%, and GC content maintained between 46.84% and 48.29% (Table 1).

**Fig. 2:**
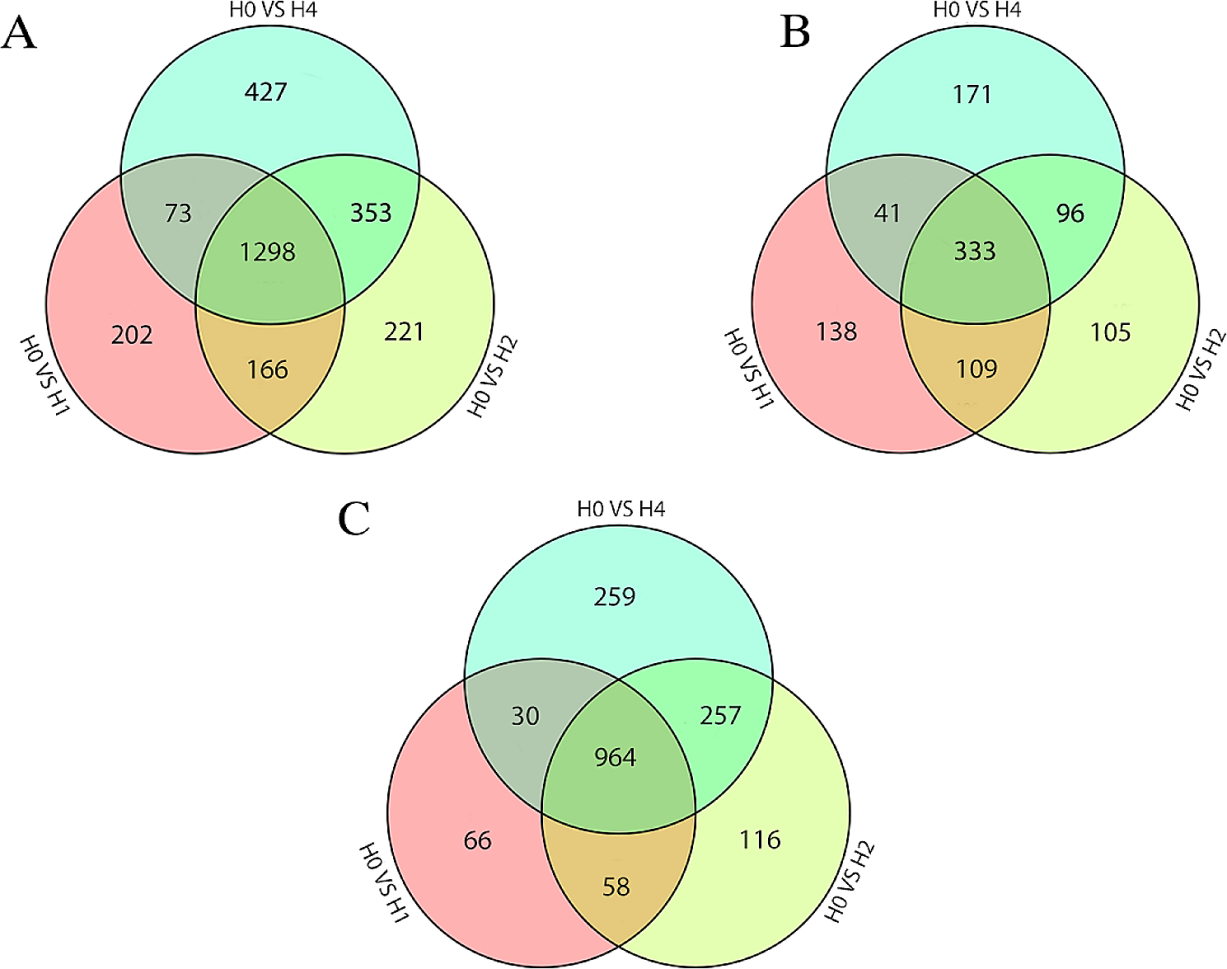
Venn diagrams of DEGs in *A. roxburghii* under different periods of high-temperature stress treatment. (**A**) All DEGs; (**B**) upregulated DEGs; (**C**) downregulated DEGs. |log2Fold Change| ≥ 2 and FDR < 0.01

Transcriptional differences between different periods of *A. roxburghii* under heat stress were determined by using pairwise comparisons of gene expression levels (H0 vs. H1, H0 vs. H2, H0 vs. H4) to identify DEGs in each comparison group. From Fig. 2, there are 1739 DEGs in H0 VS H1, of which 621 DEGs are upregulated and 1118 DEGs are downregulated. A total of 2038 DEGs were found in H0 VS H2, of which 643 DEGs were upregulated and 1395 DEGs were downregulated. A total of 2151 DEGs were found in the H0 VS H4 comparison group, of which 641 DEGs were upregulated and 1510 DEGs were downregulated. In addition, 1298 DEGs can be found overlapping in these three pairwise comparisons, of which 333 DEGs are upregulated and 964 DEGs are downregulated. These genes could be essential for *A. roxburghii*’s ability to withstand heat.

**Table 1:** Summary of transcriptome data quality in each sample

SampleID	Clean Reads	Clean Date	GC(%)	Q20(%)	Q30(%)
H0-1	22,702,492	6,795,757,956	47.48	98.03	94.34
H0-2	25,208,628	7,543,800,670	48.29	97.85	94.02
H0-3	25,242,261	7,555,174,472	47.73	97.91	94.1
H1-1	23,306,988	6,975,954,960	47.47	97.93	94.14
H1-2	24,799,217	7,423,501,606	47.09	97.81	93.8
H1-3	24,057,487	7,199,876,976	46.84	97.91	94.1
H2-1	23,966,577	7,172,904,526	47.14	98.12	94.63
H2-2	22,731,761	6,804,828,854	47.05	97.96	94.17
H2-3	25,866,829	7,742,701,880	47.04	97.64	93.46
H4-1	23,887,608	7,151,483,638	47.21	97.71	93.58
H4-2	22,373,129	6,695,979,720	47.48	97.98	94.34
H4-3	21,156,216	6,331,855,580	47.55	97.94	94.23

### GO and KEGG analyses of DEGs

**Fig. 3:**
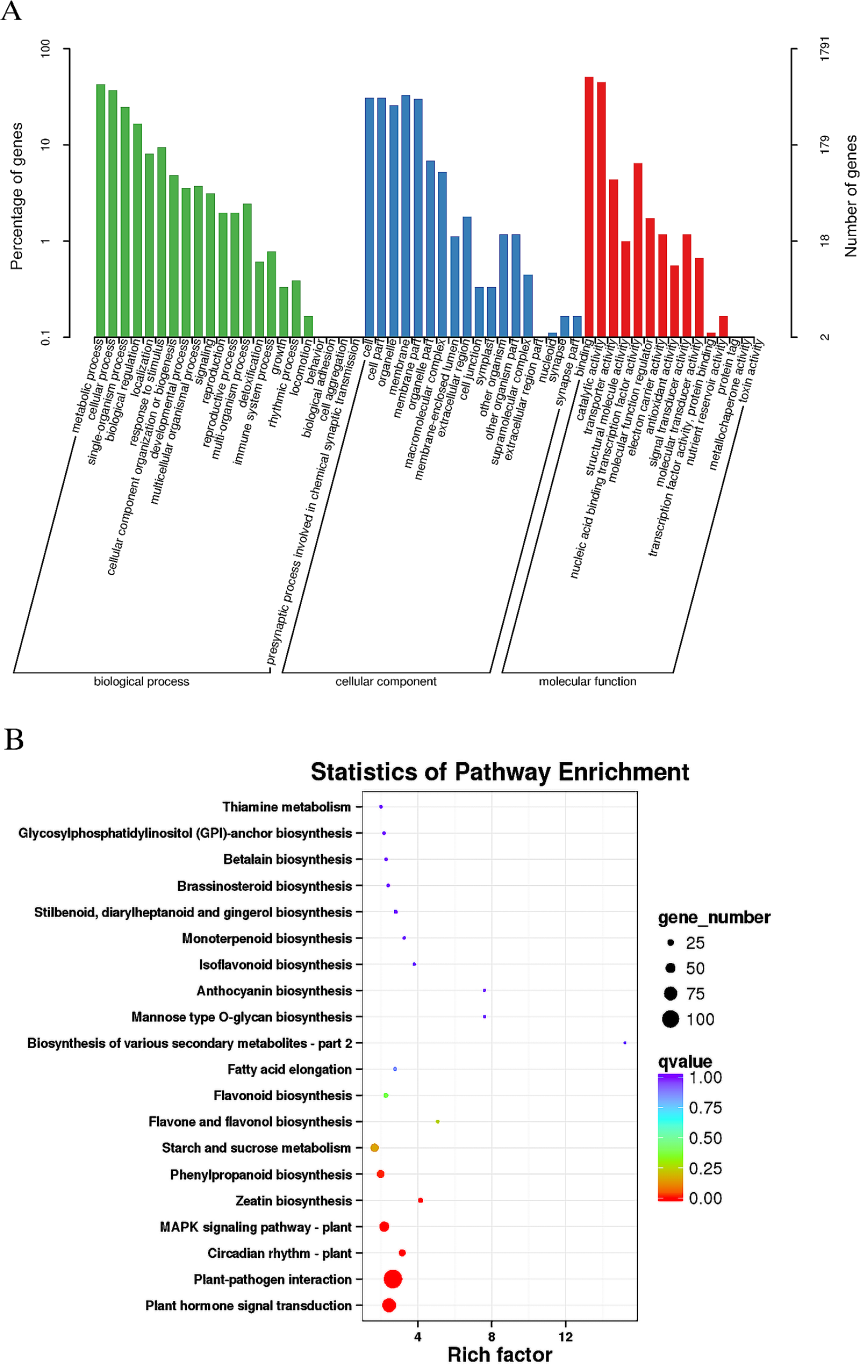
GO classification and KEGG enrichment analysis of DEGs. (**A**) GO classification of DEGs; (**B**) The 20 most enriched pathways for DEGs in KEGG

2740 DEGs in total responded to high-temperature stress. We conducted GO analysis of DEGs according to three categories: “cellular component”, “molecular function” and “biological process”. As shown in Fig. 3A, in the “biological process”, transcripts are mainly distributed in the “metabolic process” (764 DEGs), “cellular process” (666 DEGs), and “single-organism process” (448 DEGs). In the “cellular component” category, “membrane” (590 DEGs), “cell” (555 DEGs), and “cell part” (555 DEGs) were ranked highest. In the “molecular function” category, transcripts were mainly distributed in the “binding” function (911 DEGs), “catalytic activity” function (809 DEGs), and “nuclear acid binding transcription factor activity” function (137 DEGs).

We identified the top 20 main enrichment pathways by executing KEGG pathway analysis on the acquired DEGs (Fig. 3B). Remarkably, “Plant-pathogen interaction” and “Plant hormone signal transduction” were the two pathways with the greatest number of enriched genes. Therefore, we focused on these two pathways.

### DEGs involved in “Plant-pathogen interaction”

**Fig. 4:**
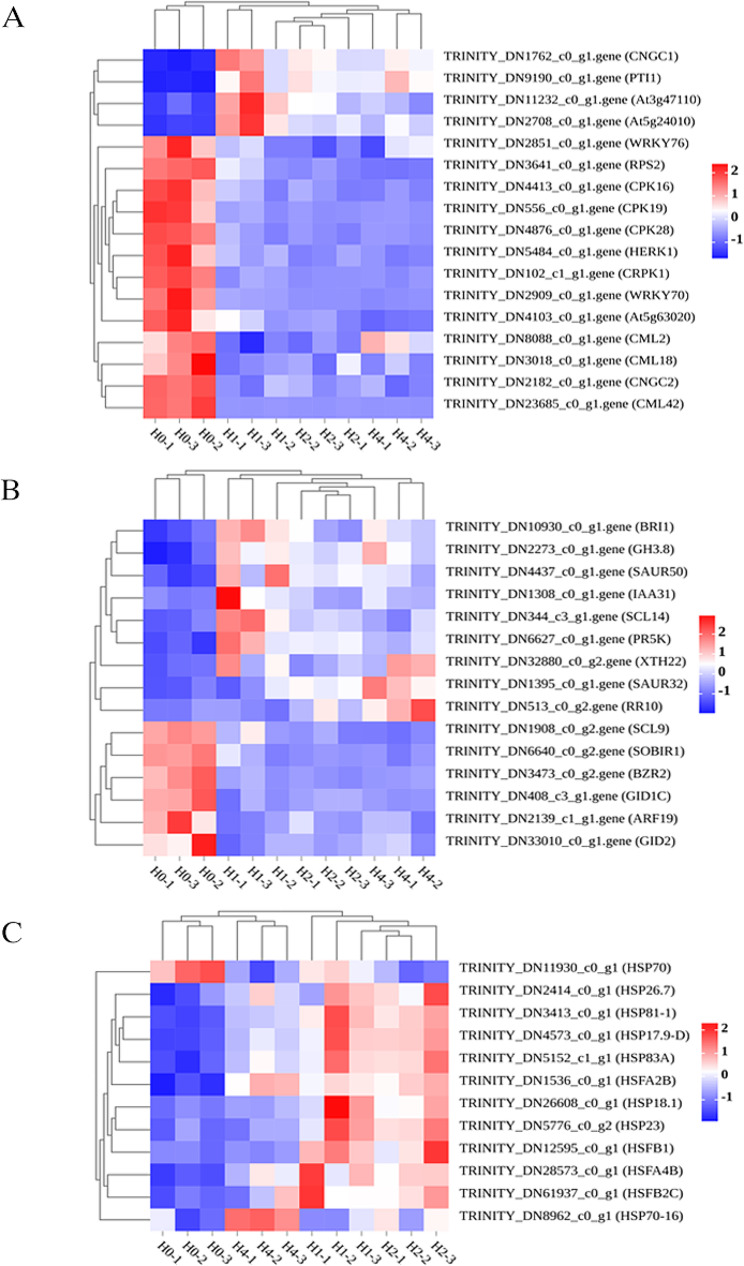
Heat map of DEGs in *A. roxburghii* under high-temperature stress. (**A**) Heat map of DEGs in the “Plant-pathogen interaction” pathway; (**B**) Heat map of DEGs in the “Plant hormone signal transduction” pathway. (**C**) Heat map of DEGs related to HSPs and HSFs

Plant-pathogen interactions trigger a signal transmission cascade that boosts the plant’s host defense system against pathogens, leading to disease resistance responses [22]. We identified 17 DEGs enriched in the “Plant-pathogen interaction” pathway (Fig. 4A). These include 2 cyclic nucleotide-gated channel (CNGC) family members *CNGC1* and *CNGC2*, 2 WRKY transcription factor family members *WRKY70* and *WRKY76*, 3 calcium-dependent protein kinase (CDPK) family members *CPK16*, *CPK19* and *CPK28*, 3 calcium-like (CML) family members *CML2*, *CML18* and *CML42*, 1 receptor-like cytoplasmic kinase9 (RLCK) family member *PTI1*, 1 leucine-rich repeat protein kinase family member *At3g47110*, 1 protein kinase family protein *At5g24010*, 1 ribosoma protein *RPS2*, 1 receptor kinases *HERK1*, 1 plasma membrane protein cold-responsive protein kinase *CRPK1*, 1 disease resistance protein (CC-NBS-LRR class) family *At5g63020*. Except for *CNGC1*, *PTI1*, *At3g47110*, and *At5g24010*, which were upregulated in high-temperature stress, all others were downregulated in expression.

### DEGs involved in “Plant hormone signal transduction”

Phytohormones have crucial functions in assisting plants in adapting to adverse environmental circumstances, and hormone signals are the perfect choice for mediating defensive responses due to their intricate network and capacity for cooperation [23]. We found 15 DEGs in the “Plant hormone signal transduction”, including 1 leucine-rich receptor-like protein kinase family protein member *BRI1*, 1 Gretchen Hagen 3 (GH3) family member *GH3.8*, 2 small auxin-up RNA (SAUR) family member *SAUR32* and *SAUR50*, 1 auxin-responsive protein IAA *IAA31*, 4 transcription factors (TF) *SCL14*, *SCL9*, *BZR2* and *ARF19*, 1 PR5-like receptor kinase *PR5K*, 1 xyloglucan endotransglucosylase/hydrolase (XTH) family member *XTH22*, 1 response regulator (RR) *RR10*, 1 receptor-like protein kinase *SOBIR1*, 2 Gibberellin (GA) receptor *GID1C* and *GID2* (Fig. 4B). *SCL9*, *SOBIR1*, *BZR2*, *GID1C*, *GID2*, and *ARF19*, six DEGs were downregulated during high-temperature stress, while the others were upregulated.

### DEGs involved in HSPs and HSFs

Heat shock proteins (HSPs) are molecular chaperones engaged in a variety of biological functions. HSPs assist in preserving cellular equilibrium by refolding misfolded proteins. When an organism experiences environmental stress, the heat shock factor (HSF) is triggered, then binds to the heat shock element (HSE) and facilitates the translation of HSP, resulting in high quantities of HSPs that shield the organism from harm [24]. Overexpression of a single HSF or HSP gene has little effect on heat tolerance, and synergistic action of HSF and HSP is required to confer high-temperature resistance in plants [6]. In this study, We identified 8 HSPs including *HSP70*, *HSP26.7*, *HSP18.1*, *HSP81-1*, *HSP17.9-D*, *HSP83A*, *HSP23*, *HSP70-16*, and 4 HSFs including *HSFB1, HSFA2B*, *HSFA4B*, and *HSFB2C* in *A. roxburghii* resistance to high-temperature stress (Fig. 4C). Among them, except for *HSP70*, which was downregulated, the rest of the DEGs were upregulated.

### Results of qRT-PCR of differentially expressed genes

**Fig. 5:**
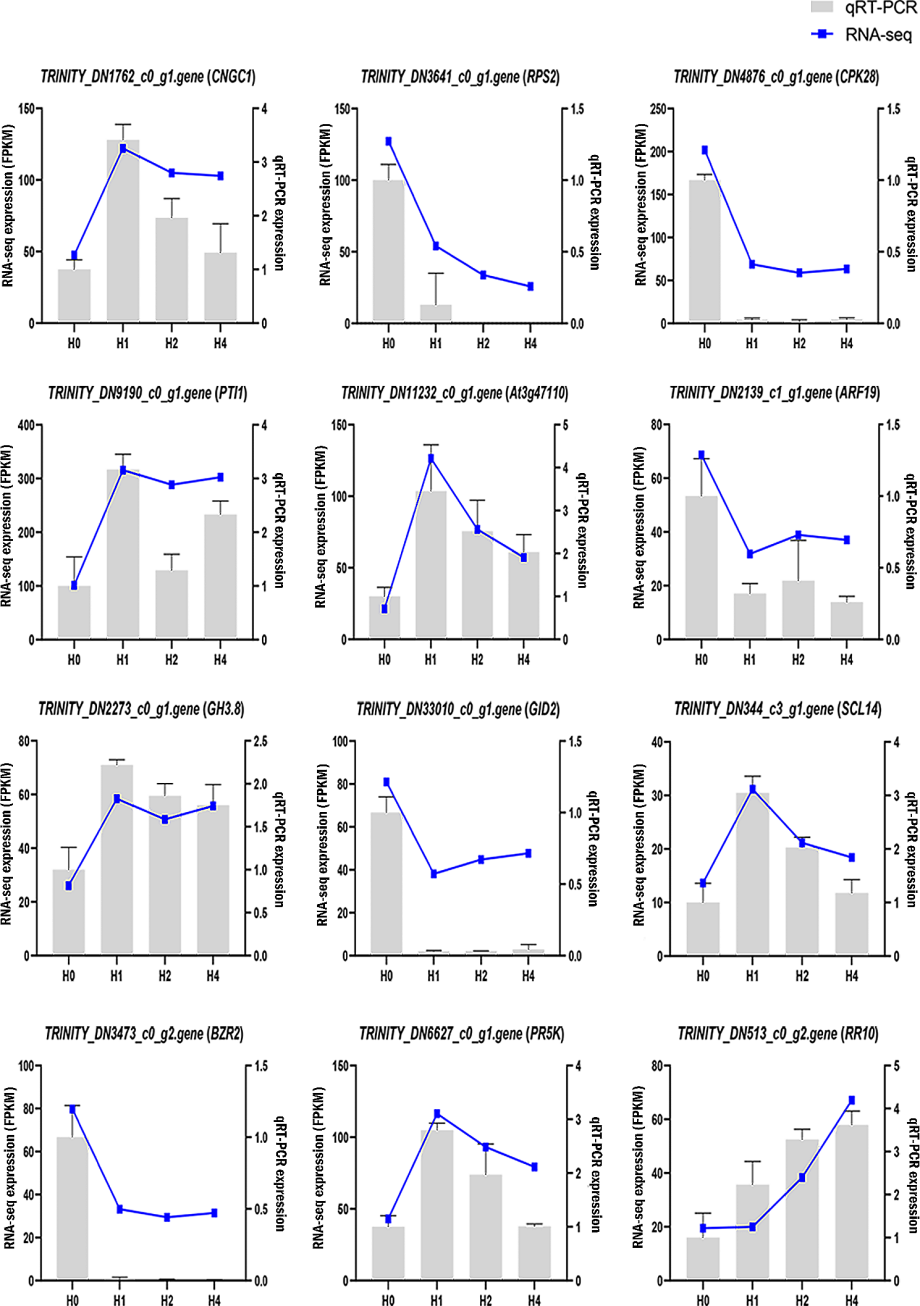
qRT-PCR validation of RNA-seq data from 12 DEGs. The qRT-PCR and RNA-seq data are presented as bar and line graphs, respectively

To verify the precision of the RNA-seq research results, we chose 12 DEGs for qRT-PCR analysis (Fig. 5). The qRT-PCR and RNA-seq expression patterns matched those found, indicating the precision of the RNA-seq analysis. For instance, the expression levels of *CNGC1*, *PTI1*, and *RR10* were notably upregulated in high-temperature stress 1, 2, and 4 h in both methods compared to the control.

## Discussion

In this experiment, high-temperature stress experiments were performed on *A. roxburghii*, physiological indicators and transcriptome were systematically studied and analyzed, and we revealed the response mode of *A. roxburghii* to high-temperature stress. We identified 2740 DEGs using RNA-seq, followed by functional annotation of DEGs and enrichment pathway annotation, and identified several major genes that might have a role in high-temperature stress.

In order to protect themselves from the harmful effects of high temperatures, plants have evolved a variety of defense mechanisms [25]. In this experiment, we investigated the changes in 9 physiological indicators of *A. roxburghii* at different times under high-temperature stress. MDA is a widely accepted biomarker of oxidative stress [26]. MDA content can indicate the degree of plant harm under various stress conditions [27]. In this research, we observed that as the duration of high-temperature stress expanded, the MDA content first reduced and subsequently increased. At 1 h of high-temperature stress, the MDA content was lower than that of the CK. We hypothesize that this is related to the scavenging of excess reactive oxygen radicals created in *A. roxburghii* and the balance of reactive oxygen metabolism during this period, which is an adaptive response demonstrated by *A. roxburghii* when exposed to 40 °C. After 1 h of high-temperature stress, MDA content increased, which indicated that prolonged high-temperature treatment damaged the protective enzyme synthesis system of *A. roxburghii* leaves, which could not scavenge excessive reactive oxygen radicals in time. Plants contain vital antioxidant enzymes like SOD, POD, and CAT, and they are involved in regulating the ROS balance in organisms to effectively reduce oxidative damage [28]. In this study, CAT activity has maintained an increasing trend, SOD activity has dropped then risen, and POD activity has decreased and then increased and then decreased. This indicates that the way antioxidant enzymes respond to temperature varies significantly depending on the kind of plant, temperature, growth stage, exposure duration, and environment [29–32]. Pro is a necessary amino acid that helps with antioxidant defense mechanisms and preserves the subcellular structure of plants [33]. In this research, Pro content first increased and then decreased, which indicated that the osmoregulatory capacity of *A. roxburghii* weakened or even began to lose under prolonged high-temperature stress. Plants’ stress tolerance can be considerably improved by the accumulation of SS [34]. In this study, the content of SS decreased rapidly at 1 h of high-temperature stress compared to the CK. However, after 1 h of high-temperature stress, to protect plants from high-temperature damage, the SS content rose as the treatment time was extended. Chlorophyll, as an important pigment of photosynthesis, has a great role in resisting stress damage [35]. The content of chlorophyll can indicate the strength of photosynthesis and the capacity of plants to tolerate extreme temperatures. SP has the functions of significantly enhancing the water-holding capacity of cells and increasing the content of bound water, which can help to maintain the osmotic potential of the internal environment and so lessen the harm resulting from stress conditions [36, 37]. In this study, we found that both chlorophyll and SP content increased first, but the content decreased after 2 h of high-temperature stress at 40 °C. This suggests that prolonged heat stress destroys the structure of chloroplasts, reduces chlorophyll content and causes severe damage to plant cells in *A. roxburghii*. In addition, we found that H_2_O_2_ content decreased between 1 h and 2 h of high-temperature stress, and we speculate that a significant amount of antioxidant enzymes may have scavenged the excess reactive oxygen radicals in time. After 2 h of high-temperature stress, the H_2_O_2_ content increased with the prolongation of the high-temperature stress time.

By analyzing the KEGG pathways involved in this experiment, we discovered that DEGs were primarily enriched in two pathways.: “Plant-pathogen interaction” and “Plant hormone signal transduction”. After further analysis, we identified 17 DEGs enriched in the “Plant-pathogen interaction”. Including *CNGC1*, *CNGC2*, *WRKY70*, *WRKY76*, *CPK16*, *CPK19*, *CPK28*, *CML2*, *CML18*, *CML42*, *PTI1*, *At3g47110*, *At5g24010*, *At5g63020*, *RPS2*, *HERK1* and *CRPK1.* CNGCs are members of the voltage-gated ion channel superfamily. Some studies have focused on the involvement of plant cyclic-nucleotide-gated channels (CNGCs) in Ca^2+^ signaling and downstream activities [38]. CNGCs are crucial for a plant’s development and response to abiotic stress [39]. In addition to directly binding and controlling target gene expression, WRKYs also function as negative regulators of adversity, contributing to the temperature stress response through various regulatory methods [40–42]. In our study, all two WRKYs were substantially downregulated in the high-temperature stress treatment, which may be related to the negative regulatory mechanisms of *A. roxburghii* or may have indirectly regulated heat stress through interactions with other transcription factors. CDPKs are involved in a large variety of physiological activities, including plant growth, and development, besides abiotic and biotic stress response [43]. It has been found that several CDPKs can either positively or negatively affect stress tolerance by ABA signaling modulation and ROS accumulation reduction [44]. Calmodulin-like (CML) proteins are the main sensors of calcium (Ca^2+^) and contribute to the control of stress responses and plant growth by transducing calcium signals into altered metabolism, transcriptional responses, or protein phosphorylation [45]. According to earlier research, *CML42* controls abiotic stress responses and insect herbivory defense [46].

15 DEGs associated with high-temperature stress are enriched in the “Plant hormone signal transduction”, including *BRI1*, *GH3.8*, *SAUR32*, *SAUR50*, *IAA31*, *SCL14*, *SCL9*, *BZR2*, *ARF19*, *PR5K*, *XTH22*, *RR10*, *SOBIR1*, *GID1C* and *GID2.* When plants encounter biotic and abiotic stresses, phytohormones, including salicylic acid (SA), ethylene (ET), jasmonic acid (JA), abscisic acid (ABA), auxin (AUX), brassinosteroids (BR), gibberellins (GA), and cytokinins (CK), orchestrate an effective defense response by activating the expression of defense genes [47]. In this study, *BRI1* and *IAA31* expression was upregulated in high-temperature stress, which may positively regulate high-temperature stress in *A. roxburghii*. *GID1C* and *GID2* were downregulated, which may be connected to the negative regulatory mechanism of *A. roxburghii*.

The mechanisms that control a plant’s growth and reaction to influences from the outside are masterly regulated by transcription factors (TFs). Plants contain approximately 58 families of transcription factors (TFs). Of these, six major TF families, AP2/ERF (APETALA2/ethylene responsive factor), bHLH (basic helix-loop-helix), MYB (myeloblastosis related), NAC (no apical meristem NAM), Arabidopsis transcription activation factor (ATAF1/2), cup-shaped cotyledon (CUC2), WRKY, and bZIP (basic leucine zipper) have been implicated in both biotic and abiotic stress responses [48]. The results indicated that four TFs, *SCL14*, *SCL9*, *BZR2*, and *ARF19*, which may have a key role in the resistance of *A. roxburghii* to high-temperature stress. *SCL14* belongs to the GRAS family of regulatory proteins. According to reports, within the GRAS family, plants’ growth, development, and response to stress are significantly regulated by the SCL9 subfamily [49]. It has been proven that BZR TFs are essential for regulating the growth of plants and are engaged in a variety of stress-response pathways [50]. Past research has demonstrated that the ARF19 protein regulates a distinct and partially overlapping collection of target genes, which is critical for auxin-mediated plant growth [51]. Among the three main families of auxin early responses, the SAUR family is one of the auxin IAA-sensitive proteins and has a variety of regulatory functions in plants [52, 53]. Previous studies have indicated that elevated temperatures trigger SAUR, which encourages plant elongation [54]. In this study, *SAUR32* and *SAUR50* were induced by high temperatures, and their expression was upregulated. These genes may be the potential key genes implicated in the response to high-temperature stress in *A. roxburghii*, but further studies on them are still needed.

In addition to the above DEGs, we also identified 8 HSPs and 4 HSFs that were differentially expressed during high-temperature stress resistance in *A. roxburghii*, including *HSP70*, *HSP26.7*, *HSP18.1*, *HSP81-1*, *HSP17.9-D*, *HSP83A*, *HSP23*, *HSP70-16*, *HSFB1*, *HSFA2B*, *HSFA4B*, and *HSFB2C.* Transcripts of the HSP70 superfamily genes are articulated during development, and this articulation is facilitated importantly in several stressful environments, suggesting that the *HSP70* gene plays a crucial role in the stress and growth process [55]. An earlier investigation revealed that *HSP70* expression was observed to be greater in the genotypes of chilies that tolerated heat than in the genotypes that failed to tolerate heat, suggesting that *HSP70* has a better protective effect against heat stress [56]. It was reported that *HSP26.7* was upregulated in response to heat stress at both seedling and flowering stages, and comparing the heat-susceptible rice cultivar Azucena to the heat-tolerant rice cultivar Co39, there was a substantial difference in the expression levels of *HSP26.7*, indicating that the capacity of rice plants to tolerate high-temperature stress was favorably connected with the expression levels of *HSP26.7* [57]. In this study, *HSP26.7* expression was upregulated in high-temperature stress, which may positively regulate high-temperature stress in *A. roxburghii.* It has been shown that *HSP70-16* is an essential gene for flower opening and mild heat response [58]. Plant HSFs are classified into three evolutionary conserved classes based on the structural characteristics of their oligomerized structural domains, A, B, and C. Class A HSFs are required for transcriptional activation. However, due to the absence of the necessary motifs made up of acidic amino acid residues, class B and C HSFs lack an activator activity [59]. It has been found that *HSFB1*, a class B member of *Arabidopsis thaliana*, negatively regulates the expression of heat-inducible Hsfs and several heat shock protein genes. On the other hand, *HSFB1* appears to be required for heat stress-induced expression of heat shock protein genes, which is required for acquired heat tolerance [60]. These genes might be significant in *A. roxburghii*’s response to high-temperature stress.

## Conclusion

In this study, physiological indexes and transcriptomics were analyzed to investigate the response mechanism of *A. roxburghii* under high-temperature stress. The results suggested that when *A. roxburghii* was exposed to high-temperature at 40 ℃ for a long time, the increase of MDA content indicated the damage of membrane. The increase of H_2_O_2_ content, the decrease of SP content and POD enzyme activity suggested the damage of cells, and the decrease of chlorophyll content indicated the damage of chloroplasts. Meanwhile, heat promoted the anti-high temperature response of *A. roxburghii*. The content of osmoregulatory substances such as Pro and SS exhibited a rising trend, and the activities of antioxidant enzymes like SOD and CAT were increased. Furthermore, we discovered that “Plant-pathogen interaction” and “Plant hormone signal transduction” pathways play important roles in the high-temperature tolerance of *A. roxburghii* and screened relevant key genes from them. In summary, these investigations showed that the molecular network mediating high-temperature stress tolerance in *A. roxburghii*, expanded our knowledge of the molecular and physiological responses of *A. roxburghii* to high-temperature stress, and contributed to the genetic improvement of high-temperature tolerance of *A. roxburghii*.

## Data Availability

The datasets generated during the current study are available in the NCBI SRA repository (https://www.ncbi.nlm.nih.gov/sra/) and the accession number is PRJNA1103245.
